# The prognostic value of interim and end-of-treatment PET/CT in patients with newly diagnosed peripheral T-cell lymphoma

**DOI:** 10.1038/bcj.2016.2

**Published:** 2016-02-12

**Authors:** J S Ham, S J Kim, J Y Choi, S H Hyun, S-K Choi, H S Kim, S H Lim, J Y Lee, S-H Jung, Y H Ko, W S Kim

**Affiliations:** 1 Division of Hematology and Oncology, Department of Medicine, Samsung Medical Center, Sungkyunkwan University School of Medicine, Seoul, Korea; 2 Department of Nuclear Medicine, Samsung Medical Center, Sungkyunkwan University School of Medicine, Seoul, Korea; 3 Department of Biostatistics and Clinical Epidemiology Center, Samsung Medical Center, Seoul, Korea; 4 Department of Pathology, Samsung Medical Center, Sungkyunkwan University School of Medicine, Seoul, Korea

The treatment outcome of cyclophosphamide, doxorubicin, vincristine and prednisolone (CHOP) as first-line chemotherapy in patients with peripheral T-cell lymphoma (PTCL) is still unsatisfactory.^1, 2^ Because the poor outcome of PTCL is mainly associated with relapse after treatment, an exact assessment of tumor viability after treatment is essential for predicting treatment failure in PTCL.^3^ For positron emission tomography–computed tomography (PET/CT) analysis, a visual analysis using a five-point scale (5-PS) was adopted as the preferred reporting method at the First International Workshop on Interim PET Scan in Lymphoma, which took place in 2009 in Deauville, France.^4^ The 5-PS is recommended for reporting PET/CT: scores of 1 and 2 represent complete metabolic response (CMR), but the clinical meaning of a score of 3 remains unclear.^5, 6^ There are limited data regarding the predictive role of the 5-PS score on interim PET/CT and end-of-treatment (EOT) PET/CT in PTCL.^7, 8, 9, 10, 11^ Moreover, the cutoff point for the 5-PS score remains unclear. Thus, we examined the treatment outcomes of patients with newly diagnosed PTCL according to their 5-PS scores for interim PET-CT and EOT PET/CT. We analyzed the prognostic value of the 5-PS score for PET/CT and determined the cutoff point that seemed to be the best predictor of outcome.

We used the WHO classification to analyze newly diagnosed PTCL patients who enrolled in one of two prospective cohorts of all consecutive patients at the time of diagnosis: the Samsung Medical Center Lymphoma Cohort Study (ClinicalTrials.gov identifier: NCT00822731) between September 2008 and December 2011, or the Samsung Medical Center Lymphoma Cohort Study II (ClinicalTrials.gov identifier: NCT01877109) between January 2012 and April 2014. Among these patients, we selected participants based on the following inclusion criteria: newly diagnosed with PTCL without a history of treatment for lymphoma; treated with CHOP every 21 days as an induction treatment; data available about the results of an interim PET/CT that was performed after the second or third chemotherapy cycle. A pretreatment PET/CT scan was performed at diagnosis before starting chemotherapy, and an interim PET/CT scan was performed after the second or third cycle of chemotherapy. The EOT PET/CT was the final PET/CT obtained at the end of the planned six cycles of CHOP. Treatment failure was defined as disease progression at any time or relapse after complete remission. The primary end point was event-free survival (EFS), which was defined as the time from the date of diagnosis to the date of documented disease progression or death from any cause, or the date of the last follow-up visit for living patients or those who dropped out. We considered dropout as an event. The secondary end point was overall survival (OS), which was defined as the time from the date of diagnosis to the date of death from any cause or the date of the last follow-up visit for living or censored patients. The Institutional Review Board of Samsung Medical Center approved this study and waived the requirement for signed informed consent.

The PET/CT protocol and criteria for positivity at the initial PET/CT scan were previously described in detail.^12^ Two experienced nuclear medicine physician (SHH and JYC) reviewed all the cases and determined the 5-PS score of the lesions. The scorers were blinded to the patients' clinical information. The 5-PS for interim PET/CT nd EOT PET/CT were analyzed for association with OS and EFS. Patients were partitioned into groups by PET/CT 5-PS scores 1–2 vs 3–5 or 1–3 vs 4–5, and the OS and EFS values of the two groups were compared using the log-rank test. These analyses were conducted using SAS version 9.4 (SAS Institute, Cary, NC, USA) and R 3.1.1 (Vienna, Austria; http://www.R-project.org).

Of the 135 patients with PTCL in our lymphoma cohort, we excluded 14 patients who were previously treated, 13 patients who were not treated with CHOP regimen and 19 patients who were ineligible for evaluation of interim PET/CT. Finally, 89 patients were analyzed using interim PET/CT scores. The clinical characteristics of the 89 patients are summarized in Table 1. Although all these patients received CHOP chemotherapy as an induction treatment, approximately three-quarters of the patients (*n*=68) completed the planned six cycles of CHOP and underwent EOT PET/CT. Among the 89 patients who underwent interim PET/CT, the disease progressed in 14 patients before completion of 6 cycles of CHOP chemotherapy, and 7 patients dropped out because of chemotherapy intolerance, death due to infection or no evaluable EOT PET/CT. Among the 89 patients who had analyzable interim PET/CT, the median follow-up duration was 20.0 months (interquartile range: 12.1–42.4 months), and 17 patients were treated with hematopoietic stem cell transplantation. Autologous stem cell transplantation was performed in 16 patients (up-front transplantation after CHOP treatment: 9 patients, residual disease after CHOP treatment: 2 patients, after recurrence: 5 patients), and only 1 patient received allogeneic stem cell transplantation because of myelodysplastic syndrome.

The patients were grouped based on two score patterns as follows: interim PET/CT 5-PS score 1–2 vs score 3–5 (cutoff value for mediastinal uptake) and score 1–3 vs score 4–5 (cutoff value for liver uptake). We analyzed OS and EFS using these two grouping patterns in 89 interim PET/CT evaluable patients (Figure 1). The 3-year EFS and OS rates for interim PET/CT-positive patients were significantly shorter than those for interim PET/CT-negative patients. Similarly, EFS and OS were also analyzed in 68 EOT PET/CT evaluable patients (Figure 1). The 3-year EFS and OS rates of EOT PET-positive patients were also significantly shorter than those of other patients. In the analysis of interim PET/CT, score groupings of 1–2 and 3–5 seemed to be better predictors of prognosis, while score groupings of 1–3 and 4–5 were better predictors in the analysis of EOT PET/CT scores.

In addition, OS and EFS were analyzed using a combination of scores for interim PET/CT and EOT PET/CT scans. The patients were divided into three groups according to 5-PS scores for interim PET/CT and EOT PET/CT as follows: interim PET negative and EOT PET negative (early responders), interim PET positive and EOT PET negative (late responders), and EOT PET positive regardless of interim PET status (non-responders). The definition of PET positivity used two cutoff values: mediastinal uptake cutoff and liver uptake cutoff. The 3-year EFS and OS rates of early responders were significantly longer than others (with scores 1–2 vs 3–5: the 3-year OS rates were 100%, 71.0%, and 62.5% in early responders, late responders and non-responders, respectively (*P*=0.0172); the 3-year EFS rates were 65.3%, 38.1% and 33.2% in early responders, late responders and non-responders, respectively (*P*=0.0419). With scores 1–3 vs 4–5: the 3-year OS rates were 94.7%, 74.1% and 42.4% in early responders, late responders and non-responders, respectively (*P*=0.0002); the 3-year EFS rates were 55.4%, 51.8% and 17.2% in early responders, late responders and non-responders, respectively (*P*=0.0359)).

The 5-PS score based on internal control is expected to be more applicable for the management of lymphoma patients. Nevertheless, the prognostic value of the 5-PS scores for PET/CT after treatment is still not clear in patients with PTCL. In this study, we evaluated the prognostic role of interim PET/CT and EOT PET/CT 5-PS scores. We chose this approach because the assessment of lesions based on the Deauville criteria has been reported to be feasible and reproducible, with good inter-observer agreement.^13^ Evaluation of the 5-PS score for interim FDG PET/CT is helpful for monitoring during ongoing treatment. Score 3 of interim PET/CT showed bad prognosis, but score 3 of EOT PET/CT showed good prognosis. In EOT PET/CT analysis, there was selection bias because many patients with early progression or complications were excluded. Two previous studies have investigated up-front autologous stem cell transplantation in PTCL.^14, 15^ In these studies, the 3-year or 5-year OS was ~50%, and the disease-free or progression-free survival was also ~50%. The prognosis for early responders was similar to or better than that for patients undergoing up-front autologous stem cell transplantation. This result was maintained among the patients except who were treated with up-front autologous stem cell transplantation. Therefore, the role of up-front autologous stem cell transplantation should be reconsidered, especially in early responders with PTCL who have good responses to CHOP chemotherapy as assessed by interim PET/CT and EOT PET/CT.

In conclusion, PTCL patients with a 5-PS score of 3 were a mixed group with both good and bad prognoses. The patients with an interim PET/CT 5-PS score of ⩾3 seemed to have a bad prognosis; therefore, it is suggested that intensified chemotherapy is needed because the classic treatment regimen might be insufficient. Therefore, we expect that an early conversion to intensified chemotherapy for PTCL will improve survival outcomes. However, patients with an EOT PET/CT 5-PS score of 3 seemed to have a good prognosis because of the selected elimination of the patients with a bad prognosis. Further studies are needed to confirm the best chemotherapy regimen for PTCL patients.

## Figures and Tables

**Figure 1:**
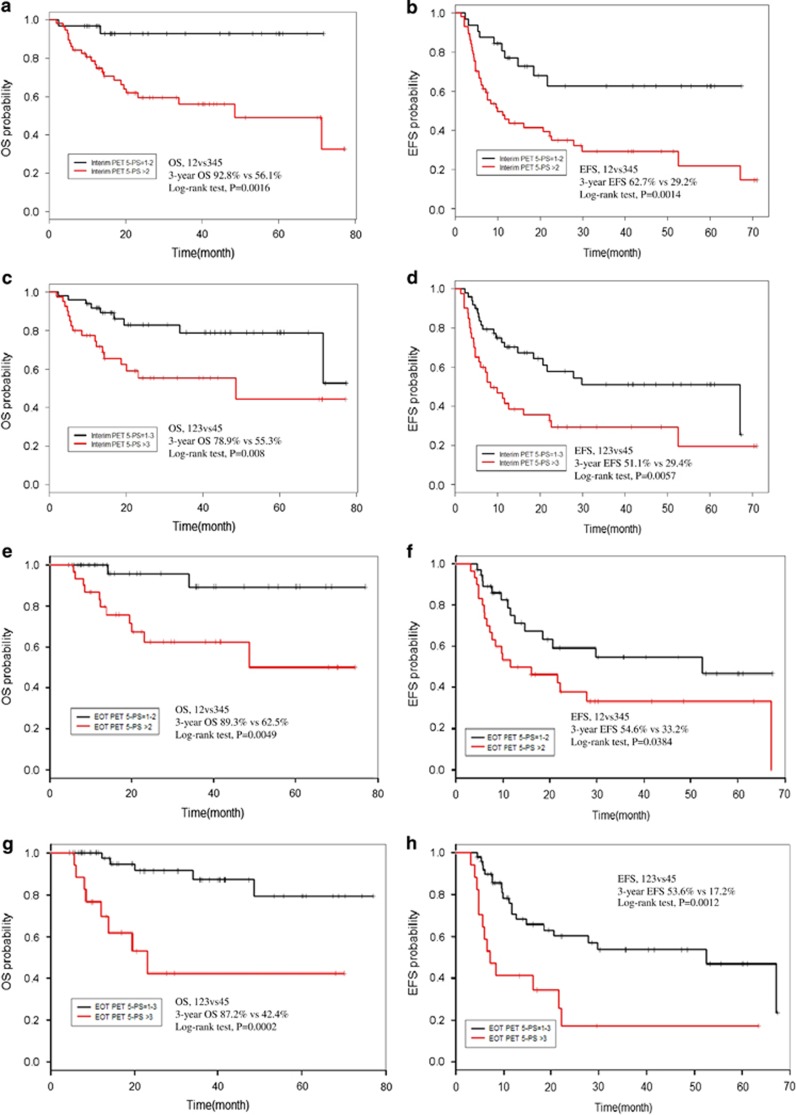
Comparison of (**a** and **c**) overall survival and (**b** and **d**) event-free survival in peripheral T-cell lymphoma patients according to a 5-point scale score of interim PET. PET positivity was defined using (**a** and **b**) mediastinal uptake (5-point scale score from 3–5) or (**c** and **d**) liver uptake (5-point scale score from 4–5) as a cutoff point. Comparison of (**e** and **g**) overall survival and (**f** and **h**) event-free survival of peripheral T-cell lymphoma patients according to a 5-point scale score of end-of-treatment (EOT) PET. PET positivity was defined using (**e** and **f**) mediastinal uptake (5-point scale score from 3–5) or (**g** and **h**) liver uptake (5-point scale score from 4–5) as a cutoff point. For scores 1–2 (*n*=32) vs 3–5 (*n*=57) on interim PET/CT 5-PS analysis, (**a**) the 3-year OS was 92.8% with scores 1–2 and 56.1% for scores 3–5 (*P*=0.0016), (**b**) and the 3-year EFS was 62.7% for scores 1–2 and 29.2% for scores 3–5 (*P*=0.0014). For scores 1–3 (*n*=49) vs 4–5 (*n*=40) on interim PET/CT 5-PS analysis, (**c**) the 3-year OS was 78.9% for scores 1–3 and 55.3% for scores 4–5 (*P*=0.008), (**d**) and the 3-year EFS was 51.1% for scores 1–3 and 29.4% for scores 4–5 (*P*=0.0057). For scores 1–2 (*n*=38) vs 3–5 (*n*=30) on EOT PET/CT 5-PS analysis, (**e**) the 3-year OS was 89.3% for scores of 1–2 and 62.5% with scores from 3–5 (*P*=0.0049), (**f**) while the 3-year EFS was 54.6% for scores 1–2 and 33.2% with scores from 3–5 (*P*=0.0384). For scores 1–3 (*n*=51) vs 4–5 (*n*=17) on EOT PET/CT 5-PS analysis, (**g**) The 3-year OS was 87.2% for scores from 1–3 and 42.4% for scores from 4–5 (*P*=0.0002), (**h**) and the 3-year EFS was 53.6% for scores from 1–3 and 17.2% for scores from 4–5 (*P*=0.0012).

**Table 1:** Baseline characteristics of interim PET/CT evaluable patients (*n*=89)

*Characteristics*	*No. of patients*	*%*
*Age (years)*		
⩽60	57	64
>60	32	36

*Sex*		
Male	54	61
Female	35	39

*Performance status*		
ECOG 0/1	73	82
ECOG ⩾2	16	18

*Serum LDH*		
Normal	39	44
Increased	50	56

*B symptoms*		
Absent	47	53
Present	42	47

*Histology*		
PTCL not otherwise specified	36	40
AITL	29	33
ALCL ALK−	9	10
ALCL ALK+	9	10
ALCL unknown ALK	1	1
Others^a^	5	6

*Ann Arbor stage*		
I	5	6
II	9	10
III	29	33
IV	46	52

*Bone marrow involvement*		
No	64	72
Yes	25	28

*IPI*		
Low	27	30
Low-intermediate	17	19
Intermediate-high	30	34
High	15	17

Abbreviations: AITL, angioimmunoblastic T-cell lymphoma; ALCL, anaplastic large cell lymphoma; ECOG, Eastern Cooperative Oncology Group; IPI, International Prognostic Index; LDH, lactate dehydrogenase; PET/CT, positron emission tomography/computed tomography; PTCL, peripheral T-cell lymphoma.

a Consisted of each pathologic diagnosis as follows: cutaneous T-cell lymphoma, enteropathy-associated T-cell lymphoma, primary cutaneous gamma-delta T-cell lymphoma, subcutaneous panniculitis-like T-cell lymphoma, mature T-cell lymphoma.
